# Pyruvate Kinase Triggers a Metabolic Feedback Loop that Controls Redox Metabolism in Respiring Cells

**DOI:** 10.1016/j.cmet.2011.06.017

**Published:** 2011-09-07

**Authors:** Nana-Maria Grüning, Mark Rinnerthaler, Katharina Bluemlein, Michael Mülleder, Mirjam M.C. Wamelink, Hans Lehrach, Cornelis Jakobs, Michael Breitenbach, Markus Ralser

**Affiliations:** 1 Max Planck Institute for Molecular Genetics, Ihnestrasse 73, 14195 Berlin, Germany; 2 Department of Cell Biology, University of Salzburg, Hellbrunnerstrasse 34, 5020 Salzburg, Austria; 3 Metabolic Unit, Department of Clinical Chemistry, VU University Medical Center, De Boelelaan 1117, 1081 HV Amsterdam, The Netherlands; 4 Cambridge Systems Biology Centre, University of Cambridge, Sanger Building, 50 Tennis Court Road, Cambridge CB2 1GA, UK; 5 Department of Biochemistry, University of Cambridge, Sanger Building, 50 Tennis Court Road, Cambridge CB2 1GA, UK

## Abstract

In proliferating cells, a transition from aerobic to anaerobic metabolism is known as the Warburg effect, whose reversal inhibits cancer cell proliferation. Studying its regulator pyruvate kinase (PYK) in yeast, we discovered that central metabolism is self-adapting to synchronize redox metabolism when respiration is activated. Low PYK activity activated yeast respiration. However, levels of reactive oxygen species (ROS) did not increase, and cells gained resistance to oxidants. This adaptation was attributable to accumulation of the PYK substrate phosphoenolpyruvate (PEP). PEP acted as feedback inhibitor of the glycolytic enzyme triosephosphate isomerase (TPI). TPI inhibition stimulated the pentose phosphate pathway, increased antioxidative metabolism, and prevented ROS accumulation. Thus, a metabolic feedback loop, initiated by PYK, mediated by its substrate and acting on TPI, stimulates redox metabolism in respiring cells. Originating from a single catalytic step, this autonomous reconfiguration of central carbon metabolism prevents oxidative stress upon shifts between fermentation and respiration.

## Graphical abstract

**Graphical Abstract:**

**Figure:**
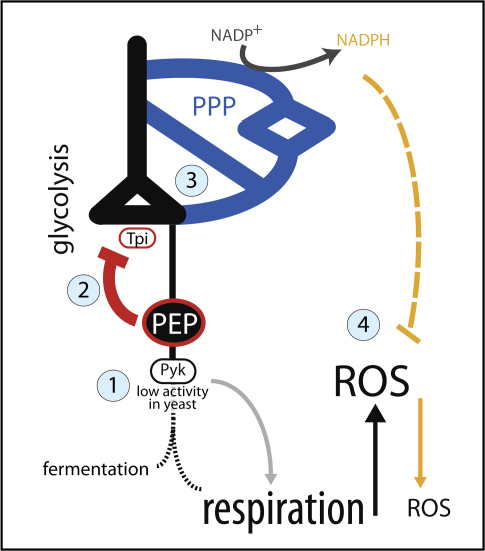

**Highlights:**

► Low pyruvate kinase (PYK) activity activates respiration in yeast ► Accumulation of the PYK substrate PEP inhibits the glycolytic enzyme TPI ► TPI inhibition stimulates the pentose phosphate pathway, preventing ROS accumulation ► The PYK-PEP-TPI feedback loop synchronizes respiration and redox metabolism

## Introduction

Glycolysis and oxidative phosphorylation are primary sources of cellular energy. Pyruvate, the end product of glycolysis, is either metabolized through the citrate cycle and respiratory chain or fermented to lactate or ethanol. Eukaryotic cells depend on glycolysis but can grow without oxidative phosphorylation. In the first half of the twentieth century, Otto Warburg and his coworkers observed that cells switch from oxidative to fermentative metabolism during tumorigenesis. Despite the presence of oxygen, most cancer tissue respires with low efficiency but has increased glucose consumption and lactate secretion (Hsu and Sabatini, 2008; Levine and Puzio-Kuter, 2010; Najafov and Alessi, 2010; Warburg, 1956). Recently, it has become clear that this metabolic transition is not specific to cancer cells but rather a common metabolic feature of cells that rapidly proliferate. Warburg-like effects have been described in yeast (Ruckenstuhl et al., 2009), during T cell proliferation (Colombo et al., 2010), and upon reprogramming fibroblasts into IPS cells (Prigione et al., 2010).

The glycolytic enzyme pyruvate kinase (PYK) which catalyzes the conversion of phosphoenolpyruvate (PEP) to pyruvate has been implicated in the regulation of the Warburg effect. The low-active splice form of the PKM-type pyruvate kinase (PKM2) is present at higher concentration in cancers compared to matched control tissue (Bluemlein et al., 2011; Christofk et al., 2008). A change to expression of the higher active PKM1 using a lentiviral system slowed cancer progression in xenograft models (Christofk et al., 2008). Recent results indicate that PKM2 is regulated by phosphorylation of tyrosine residue 105. This modification inhibited the formation of the active PKM tetramer in cancer cells (Hitosugi et al., 2009). Furthermore, PKM2 hydroxylation on proline 403/408 stimulates binding and activation of hypoxia-induced factor HIF1α, increasing the expression of metabolic enzymes under hypoxia in a PYK activity-independent manner (Luo et al., 2011).

Respiration and fermentation differ in their metabolic consequences. Although highly efficient, oxidative phosphorylation produces a significant amount of reactive oxygen species (ROS). Under physiological conditions, up to 1%–2% of metabolized oxygen is converted to superoxide (Cadenas and Davies, 2000). To avoid an excess of oxidizing compared to reducing molecules in the cell (oxidative stress), ROS are removed by a complex machinery. ROS neutralization, and maintenance of the redox balance, involves shuttling of reduction power through the pyridine nucleotide NADPH. NADPH serves as cofactor for fatty acid synthesis, and the recycling steps within the glutathione, thioredoxin, and peroxiredoxin systems, whose redox state control is crucial (Pollak et al., 2007; Ying, 2008).

Here we describe a mechanism that synchronizes redox metabolism when respiration is activated, and show that both respiration and the production of redox equivalents are synchronized by PYK. We engineered yeast strains with different PYK activities and discovered that yeast respiration is inversely correlated with PYK enzyme activity. However, an increase in ROS levels was not detected when respiration was activated, and in addition, resistance to oxidants increased.

We found that a metabolic feedback loop is responsible for preventing an increase in ROS upon respiration activation. Low PYK enzyme activity caused accumulation of PEP, its substrate, which in turn inhibited TPI, an enzyme of upper glycolysis. This inhibition of TPI increased metabolite content of the pentose phosphate pathway (PPP), a catabolic pathway closely connected to glycolysis. PPP is an important source of reduced NADPH (Slekar et al., 1996; Wamelink et al., 2008) and is involved in the adaptation of gene expression during stress conditions (Krüger et al., 2011). In yeast strains deficient for this process, ROS were improperly cleared, accumulated, and caused damage on macromolecules when cells started respiration. Thus, metabolic feedback activation of the PPP prevents oxidative stress upon induction of oxidative metabolism. Both processes are concordantly regulated by the same enzyme, PYK, a new hub in the regulation of these fundamental metabolic processes.

## Results

### Generation of Yeast Strains with Varying PYK Activity

**Figure 1:**
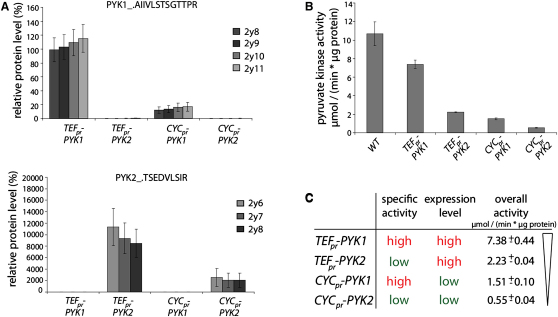
A Yeast Model for Studying Pyruvate Kinase (A) PYK expression levels. Quantification of a Pyk1p (upper panel)- or Pyk2p (lower panel)-specific peptide in *Δpyk1Δpyk2* yeast expressing *TEF_pr_-PYK1*, *TEF_pr_-PYK2*, *CYC_pr_-PYK1*, or *CYC_pr_-PYK2*. Normalized peak intensities of >3 MRM transitions are presented as the relative expression level (%) compared to the concentrations in the wild-type strain BY4741. For both isoforms, levels from the *CYC1* promoter (*CYC_pr_*) were around 20% of the expression levels from the *TEF1* (*TEF_pr_*) promoter. Error bars, ±SD from normalization to three reference peptides. (B) Pyruvate kinase activity of BY4741 (WT) and *TEF_pr_-PYK1*, *TEF_pr_-PYK2*, *CYC_pr_-PYK1*, or *CYC_pr_-PYK2* yeast. Error bars, ±SD. (C) Overview on the yeast models with varying PYK activity.

Yeast possesses two PYK paralogues (PYK1, PYK2) which are differentially expressed between fermentative and oxidative metabolism (Boles et al., 1997). By expressing either Pyk1p or Pyk2p under the control of either a strong (*TEF1*) or a weak (*CYC1*) constitutive promoter in yeast strains deleted for both endogenous loci, we generated four yeast strains with different PYK activities. Expression of Pyk1p and Pyk2p was quantitated using liquid chromatography/multiple reaction monitoring (LC-MRM) by four MRM (Q1/Q3) transitions as described (Bluemlein and Ralser, 2011). *PYK1* expression driven by the *TEF1* promoter was at a similar level as endogenous *PYK1* expression level in the wild-type strain (2.9 × 10^5^ copies per cell) (Ghaemmaghami et al., 2003) (Figure 1A). Consistent with this, *PYK2* (expressed at 2.13 × 10^3^ copies in wild-type cells [Ghaemmaghami et al., 2003]) exceeded the concentration when expressed under the *TEF1* promoter (Figure 1A, lower panel). In both cases, the *CYC1* promoter constructs expressed at ∼20% of this level.

Then we determined the enzymatic activity of PYK in these strains. *TEF1_pr_-PYK1* yeast had 69% of wild-type activity, *CYC1_pr_-PYK1* yeast 14%. As expected, activity in PYK2-expressing cells was lower: the *TEF1_pr_-PYK2* strain had 22% and the *CYC_pr_-PYK2* strain 5% activity (Figure 1B). Thus, protein quantification and enzyme activity measurements confirmed that, in wild-type yeast, *PYK2* is expressed at lower levels compared to *PYK1* and has a lower specific activity. Controlled expression of these proteins with two promoters of different strength generated four strains with gradually decreasing PYK activity (Figure 1C).

### Low Pyruvate Kinase Activity Activates Respiration

**Figure 2:**
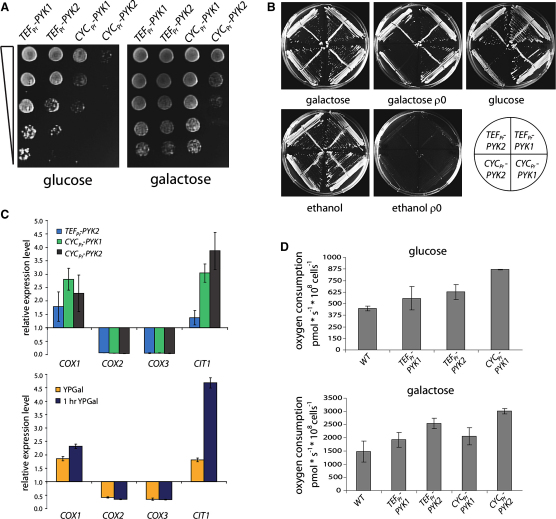
PYK Activity Regulates Respiration (A) Growth deficits caused by low PYK activity are rescued on galactose. Strains were grown overnight, diluted to an OD_600_ of 3.0, and spotted as serial dilutions (1:1, 1:5, 1:25, 1:125, and 1:625) on YPD (glucose) and YPGal (galactose). (B) Galactose rescue of low PYK activity requires a functional respiratory chain. Yeast strains with deficient mitochondrial DNA (ρ0) were grown alongside with controls (ρ+) on YP media with the indicated carbon sources. Galactose did not compensate for the growth deficits caused by low PYK activity in ρ0 yeast. (C) Regulation of oxidative metabolism's mRNA expression by PYK and galactose. *COX1*, *COX2*, *COX3*, and *CIT1*, implicated in oxidative energy metabolism, were analyzed by qRT-PCR. Expression values are given as fold change compared to *TEF_pr_-PYK1* yeast, relative to glucose grown yeast (upper panel). Changes in mRNA expression of the same transcripts in yeast shifted to galactose for 1 hr, or permanently, activating oxidative metabolism (lower panel). Both conditions caused a similar mRNA expression fingerprint. Error bars, ±SD. (D) Oxygen consumption increases with low PYK activity. Oxygen consumption in logarithmically growing wild-type yeast and PYK mutants was determined on glucose (upper panel) and galactose media (lower panel), and cell numbers were determined with an electric field multichannel cell counting system (CASY). Overall oxygen consumption was three times increased in galactose, low PYK activity increased oxygen uptake under both conditions. Error bars, ±SD.

Growth of these yeast strains correlated with PYK activity. *TEF_pr_-PYK2* grew slower as *TEF_pr_-PYK1*, *CYC_pr_-PYK1* even more slowly, and the strain with the lowest PYK activity (*CYC1_pr_-PYK2*) showed very slow growth only (illustrated by a spot test [Figure 2A, left panel]; phenotypes in liquid culture were similar [data not shown]).

Surprisingly, the differences in growth were largely rescued by a switch of the carbon source from glucose to galactose (Figure 2A, right panel). Both carbon sources are fermentable but have different effects on respiration. Glucose represses respiration, much more than galactose (Carlson, 1999; Ruckenstuhl et al., 2009). Therefore we investigated whether the galactose rescue was attributable to an increase in respiration.

First, we tested if galactose also rescued the growth defects in respiratory-deficient (ρ0) cells. Bona fide ρ0 strains were generated by the method of Goldring et al. (1970) and lost the capability to grow on nonfermentable carbon sources (ethanol, glycerol; *pet* phenotype), indicating mitochondrial deficiency (Figure 2B). Differences in growth between glucose and galactose media were abolished; i.e., ρ0-*CYC_pr_-PYK2* cells were not viable on galactose media and displayed a similar growth phenotype as their ρ+ counterparts on glucose (Figure 2B). Thus, galactose rescue of growth deficiencies caused by low PYK activity required a functional respiratory chain.

Next, we analyzed transcripts involved in oxidative energy metabolism (*COX1*, *COX2*, *COX3*, and *CIT1*) by qRT-PCR. Low PYK activity led to the upregulation of *COX1* and *CIT1* and the downregulation of *COX2* and *COX3* (Figure 2C, upper panel). Shifting cells from glucose to galactose had a similar effect (Figure 2C, lower panel). Thus, low PYK activity and a shift to respiratory-active media led to a similar regulation of these enzymes.

Finally, we measured oxygen uptake in a closed chamber oxygraph (Oroboros). There was a strong increase in oxygen uptake when PYK activity was low (Figure 2D, upper panel). On glucose, oxygen consumption doubled in *CYC_pr_-PYK1* yeast compared to the wild-type strain. A similar set of experiments was conducted on galactose media, which facilitated including *CYC_pr_-PYK2* yeast. Galactose increased the overall oxygen consumption ∼3-fold; but also here respiration was stimulated by PYK activity (Figure 2D, lower panel). The *CYC_pr_-PYK2* strain consumed 3005 pmol oxygen ^∗^ 10^8^ cells^−1^^∗^ s^−1^ (6-fold greater than glucose wild-type). Consequently, PYK-mediated regulation of respiration was additive to the release of glucose repression and is thus an independent process.

In summary, several experiments demonstrated an increase in oxidative metabolism when PYK activity was low. Growth differences between PYK mutants were abolished on respiratory-active galactose media, and depletion of the respiratory chain prevented this effect. Furthermore, reduced PYK activity and a release of glucose repression provoked a similar mRNA expression fingerprint on studied enzymes of oxidative metabolism. Most importantly, oxygen consumption was inversely correlated with PYK activity.

### High Respiration Rates Are Coupled to an Increased Antioxidative Capacity

**Figure 3:**
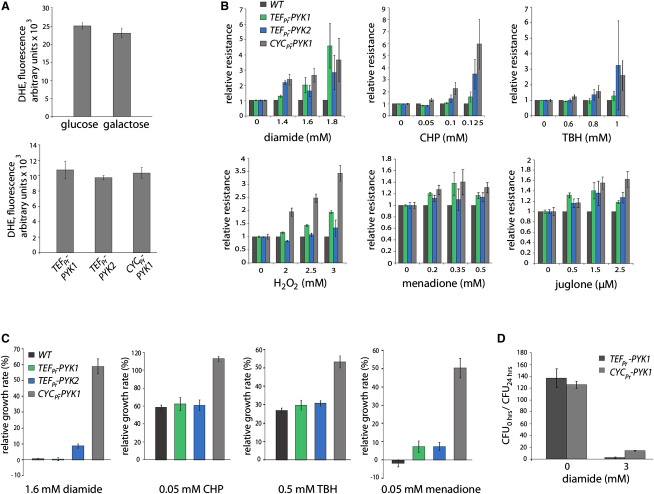
Activation of Respiration Does Not Increase ROS Levels, but It Does Increase Oxidant Resistance (A) Superoxide levels are not increased upon the activation of oxidative metabolism. ROS levels were determined by assaying DHE fluorescence in exponentially growing yeast in SCGluc and SCGal media (upper panel) or in yeast with varying PYK activity (lower panel). Error bars, ±SD. See also Figure 6B. (B) Oxidant resistances increase with low PYK activity. BY4741 and yeast strains with varying PYK activity were spotted in triplicates on media containing diamide, cumene hydroperoxide (CHP), tert-butyl hydroperoxide (TBH), hydrogen peroxide (H_2_O_2_), menadione, or juglone. Pictures taken from spot tests were analyzed with CellProfiler, values indicate the ratio of spot intensity of the indicated strain to the wild-type control. Error bars, ±SD. See also Figure S1. (C) Low PYK activity increases growth capacity in the presence of oxidants. Yeast models with different PYK activity were grown with our without oxidants and analyzed spectrophotometrically. Values indicate the doubling time relative to the nontreated control culture. Error bars, ±SD. (D) Increased survival of yeast with low PYK activity in oxidant media. *TEF_pr_-PYK1* and *CYC_pr_-PYK1* yeast was incubated in YPD and in YPD supplemented with 3 mM diamide for 24 hr, plated, and formed colonies counted. Error bars, ±SD.

Respiration is responsible for most of the macromolecule oxidation that occurs in living cells, since high amounts of ROS leak from the respiratory chain (Cadenas and Davies, 2000; Turrens, 1997). To detect overall ROS levels, we stained yeast with dihydroethidium (DHE). The DHE oxidation products 2-hydroxyethidium and ethidium are fluorescent, transferring this dye into a sensitive and reliable ROS probe, which primarily detects the superoxide anion (Benov et al., 1998). Remarkably, the 3-fold greater oxygen consumption in yeast grown on SC-galactose media (Figure 2D) did not result in an increased DHE fluorescence, which indicates that superoxide levels were unchanged (Figure 3A, upper panel). A similar result was obtained for low PYK activity: despite the strong increase in respiration, ROS levels did not increase (Figure 3A, lower panel). A staining for H_2_O_2_, the first intermediate when superoxide is neutralized through superoxide dismutase, confirmed these results (Figure 6B). Thus, respiring cells compensate for the increased ROS leakage.

We tested the influence that respiration activation had on oxidant resistance. Strains with varying PYK activity were spotted onto agar containing the oxidants H_2_O_2_, diamide, cumene hydroperoxide (CHP), tert-butyl hydroperoxide (TBH), juglone, and menadione. Growth was measured as relative spot intensities using CellProfiler (Carpenter et al., 2006) (Figure 3B). Remarkably, increased respiration of cells with low PYK activity did not sensitize them to oxidants. Indeed, resistance to diamide, CHP, and TBH was strongly increased. Similar results were obtained when respiration was activated with galactose, as tested with diamide (see Figure S1 available online). Low PYK activity also increased resistance to H_2_O_2_, juglone, and menadione, although to a lesser extend (Figure 3B). Effects of diamide, CHP, tert-butylhydroperoxide, and menadione were further tested in liquid cultures. Overnight cultures of BY4741 and the PYK mutants were diluted, supplemented with the oxidants, and their growth followed spectrophotometrically. Low PYK activity increased the resistance to these oxidants, as higher growth capacity was maintained (Figure 3C). Interestingly, also the slight difference in PYK activity between the wild-type BY4741 and the *TEF_pr_*-PYK1 strain (Figure 1) pictured as increase in oxidant resistance (Figures 3B and 3C). Finally, we tested for maintenance in colony formation in the presence of a high oxidant dose. Liquid cultures were supplemented with 3 mM diamide and plated onto YPD agar before and 24 hr after addition of the oxidant. *CYC_pr_*-*PYK1* yeast maintained a higher number of forming colonies, indicating increased survival under very strong redox stress (Figure 3D). Thus, low PYK activity triggered respiration but broadly increased oxidant resistances rather than ROS levels.

### Low PYK Activity Causes Accumulation of Its Substrate Phosphoenolpyruvate

**Figure 4:**
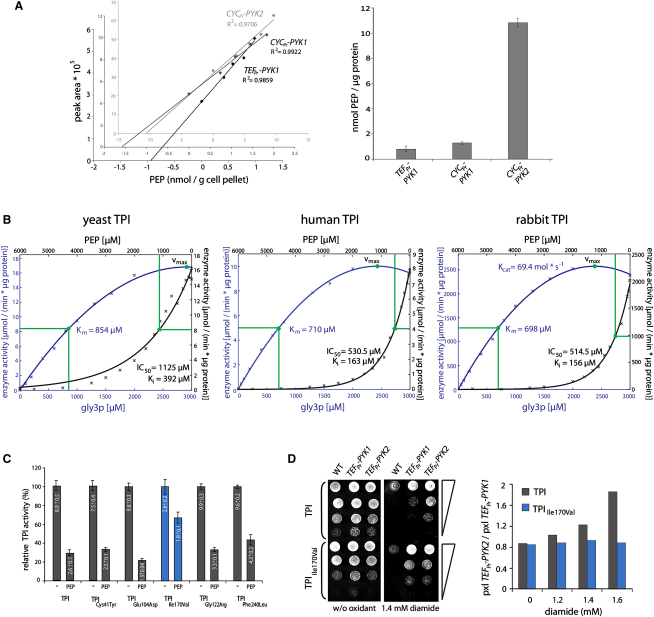
Phosphoenolpyruvate Accumulates in Cells with Low PYK Activity and Inhibits Triosephosphate Isomerase (A) HILIC-MRM quantification of PEP (left panel) Standard addition of PEP to whole-cell methanol/water yeast extracts; and quantification of PEP separated by HILIC using multiple reaction monitoring (MRM). Quantification is demonstrated by linear regression, R^2^ vales of >0.97 were obtained. (Right panel) HILIC-MRM quantification of PEP in *TEF_pr_-PYK1*, *CYC_pr_-PYK1*, and *CYC_pr_-PYK2* yeast; PEP is strongly accumulated in yeast with low PYK activity. Error bars, ±SD, n = 3. (B) PEP inactivates yeast and mammalian TPI. Michaelis-Menten kinetics were determined for yeast TPI (left panel), human TPI (middle panel), and rabbit muscle TPI (right panel). To determine v_max_ and K_m_, the TPI substrate gly3p was added in incremental doses (left y and lower x axis, blue; y values are normalized to total cellular protein [left and middle panel] or to the purified protein [right panel]). For all TPI isozymes, v_max_ is highlighted with a green dot in the saturation curve; K_m_ values are given in μM (right y and upper x axis, black). Inhibition of TPI activity demonstrated by addition of PEP in incremental concentrations; IC_50_ values and the inhibitory constant K_i_ are given in μM, the Kcat in Mol^∗^s^−1^. (C) Inactivation of pathogenic TPI alleles by PEP. TPI activity was assayed in transgenic yeast expressing human TPI (WT) or indicated pathogenic TPI alleles without or in the presence of 900 μmol PEP. Error bars, ±SD. Values within bars indicate the absolute enzyme activity in μmol/(min^∗^μg protein). (D) PYK activity does not change oxidant resistance in yeast expressing TPI_Ile170Val_. The wild-type control, transgenic yeast expressing either *PYK1* or *PYK2*, and either human TPI or human TPI_Ile170Val_ were spotted as serial dilutions onto SC media without or with diamide (left panel). Spot growth on different concentrations was analyzed with CellProfiler and normalized to the strain with highest PYK activity (right panel). Low PYK activity increased diamide tolerance if expressed in combination with human TPI, but there were no differences in TPI_Ile170Val_-expressing yeast.

To investigate whether metabolic changes were responsible for the increased oxidant resistances, we started by quantifying the PYK substrate PEP. We developed a hydrophilic interaction liquid chromatography/multiple reaction monitoring (HILIC-MRM) method suitable for quantification of this highly polar metabolite out of whole-cell extracts. PEP was extracted with methanol/water and separated on a HILIC column (1.7 μm particle size, 2.1 × 100 mm) using ultra-high-pressure binary pump (Agilent 1290) at 800–1000 bar and a flow rate of 1 ml/min. Quantification was conducted on a triple-quadrupole mass spectrometer (AB/Sciex QTRAP5500) with electrospray ionization (ESI) and achieved by standard addition, which resulted in a reliable method as solid linear correlations (R^2^ 0.97–0.99) were achieved (Figure 4A, left panel).

PEP was determined in *TEF_pr_-PYK1*, *CYC_pr_-PYK1*, and *CYC_pr_-PYK2* yeast that were pregrown overnight in YPD, diluted with fresh media to an OD_600_ of 0.15, and cultivated in triplicates for further 5 hr. Low PYK activity resulted in strong accumulation of PEP. *CYC_pr_*-PYK2 yeast had a 13.6 times higher PEP concentration as the *TEF_pr_-PYK1* strain (Figure 4A, right panel).

### PEP Is an Inhibitor of Triosephosphate Isomerase

Searching for physiological consequences of accumulating PEP, we noticed that this molecule influenced an enzyme-coupled assay of phosphofructokinase, due to inhibition of the component TPI (Fenton and Reinhart, 2009). TPI is a glycolytic enzyme that converts the three carbon sugars glyceraldehyde 3-phosphate (gly3p) and dihydroxyacetone phosphate (dhap). We examined TPI activity and determined the kinetic parameters for yeast TPI (endogenous), human TPI (expressed in yeast), and rabbit TPI (purified from muscle). Yeast TPI had a K_m_ of 854 μM, human TPI 710 μM, and rabbit TPI 698 μM (Figure 4B). Next, we tested whether PEP inhibits TPI (black axes). All TPI paralogues were efficiently inhibited by titrating PEP (Figure 4B). Using the Cheng-Prusoff equation (Cheng and Prusoff, 1973), we calculated a PEP inhibitory constant (K_i_) of 392 μM for yeast TPI, 163 μM for human TPI, and 156 μM for rabbit TPI. Thus, TPI is inactivated by physiological PEP concentrations. Human and rabbit TPI were inactivated twice as efficiently compared to yeast TPI.

### TPI Inhibition Is Required for Increased Antioxidative Capacity in Respiring Yeast

We investigated whether TPI feedback inhibition is mechanistically linked to the increase in oxidant resistance of respiring cells, as we had observed earlier that low TPI activity increases oxidative stress resistance in yeast and *C. elegans* (Ralser et al., 2007, 2009).

First, PEP inhibition was tested on five human TPI alleles that have been associated with the pathogenesis of the metabolic syndrome TPI deficiency (Orosz et al., 2009). Wild-type human TPI, TPI_Cys41Tyr_, TPI_Glu104Asp_, TPI_Gly122Arg_, and TPI_Phe240Leu_ were all strongly inhibited in the presence of 900 μM PEP. However, TPI_Ile170Val_, an allele with low catalytic activity (Ralser et al., 2006), was significantly less inhibited (Figure 4C).

The identification of the relative PEP resistance of TPI_Ile170Val_ allowed the generation of yeast strains in which TPI activity was insensitive to PEP accumulation. Double knockout mutants (Δ*pyk1*Δ*tpi1*) that expressed either human TPI or human TPI_Ile170Val_ and either *PYK1* or *PYK2* ectopically were generated and assayed for their oxidant tolerance. The strain with low *PYK2* activity gained diamide resistance when expressed in combination with wild-type human TPI. This effect was abolished when TPI_Ile170Val_ was expressed, as there were no differences in oxidant tolerance between low and high PYK activity in this strain (Figure 4D). Thus, redox-protective effects of low PYK activity were not additive to low TPI activity, and not observed in a yeast strain where TPI is insensitive to PEP inhibition.

### PPP Activation in Respiring Yeast through TPI Feedback Inhibition

**Figure 5:**
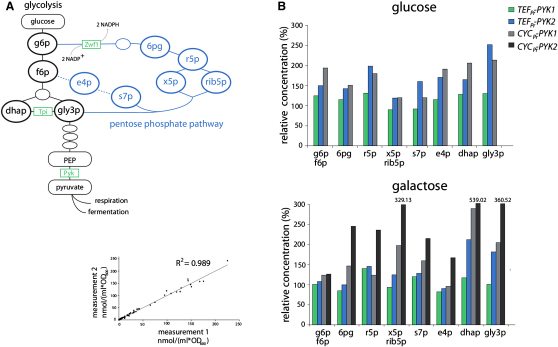
The PYK-PEP-TPI Feedback Loop Stabilizes ROS Levels by Activating the Pentose Phosphate Pathway (A) Overview on the PPP and glycolysis. (B) Cells with low PYK activity have increased concentrations of PPP intermediates. Sugar phosphates were extracted from exponential cultures and quantified by LC-MRM. All measured PPP intermediates—g6p (glucose 6-phosphate), f6p (fructose 6-phosphate), 6pg (6-phosphogluconate), r5p (ribose 5-phosphate), rib5p (ribulose 5-phosphate), x5p (xylulose 5-phosphate), s7p (sedoheptulose 7-phosphate), and e4p (erythrose 4-phosphate)—as well as TPI substrates dhap (dihydroxyacetone phosphate) and gly3p (glyceraldehyde 3-phosphate) were increased in yeast with low PYK activity grown in glucose (upper panel) and galactose media (lower panel). Reproducibility of sugar phosphate quantification is demonstrated by linear regression (R^2^ 0.989) (lower panel left). See also Table S1.

Previously, it was reported that NADP^+^ reduction, and the concentration of PPP intermediates, increases when TPI is mutant (Kleijn et al., 2007; Ralser et al., 2007). To investigate if feedback inhibition had a similar effect, we quantified PPP intermediates by LC-MRM (Wamelink et al., 2009) (Figure 5A). In cells with low PYK activity there was an increase in all PPP intermediates examined (Figure 5B, upper panel, absolute values are given as Table S1, reproducibility is demonstrated by linear regression [Figure 5B, lower left]). The TPI substrates dhap and gly3p were included in the analyses. Indicating lowered TPI activity, these two metabolites showed the strongest increase. Including the *CYC_pr_-PYK2* strain, these measurements were then performed on galactose-grown cultures (Figure 5B, lower panel). Effects were similar to glucose, although there dhap and gly3p accumulation was stronger; overall the *CYC_pr_-PYK2* with lowest PYK activity exhibited strongest changes.

### PPP Activation Prevents ROS Accumulation and Is Necessary for Increased Oxidant Tolerance of Respiring Cells

PPP splits into a nonoxidative and oxidative branch, of which the latter is responsible for the reduction of NADP^+^ to NADPH, and not reversible. Therefore, deletion of its first enzyme, glucose 6-phosphate dehydrogenase (*Zwf1p*), separates the oxidative PPP from glycolysis and prevents its function as NADPH donor (Wamelink et al., 2008).

**Figure 6:**
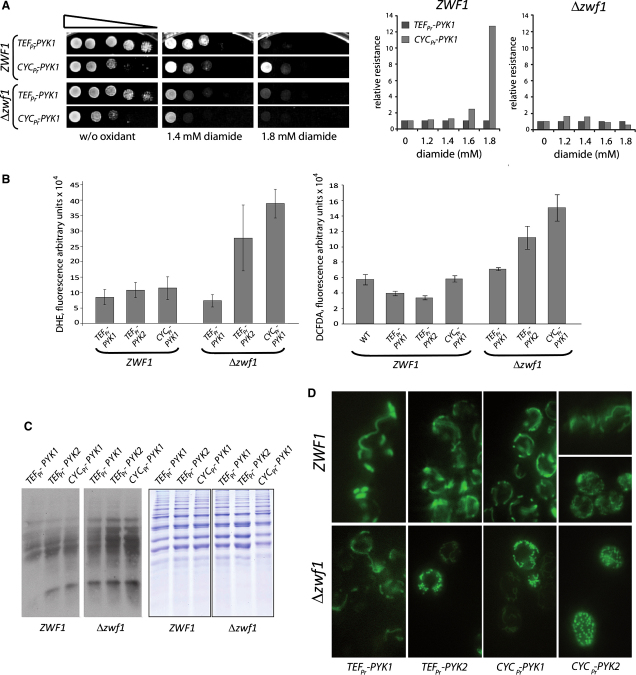
The PYK-PEP-TPI Feedback Loop Protects Cells from ROS-Induced Damage during Respiration (A) Low PYK activity in *Δzwf1* yeast does not protect against oxidants. The first enzyme involved in the irreversible NADPH producing oxidative PPP branch (*ZWF1*) was deleted, and PYK strains were tested for diamide resistance (left panels). Densitometric processing of (A), including more oxidant concentrations (right panels). Deletion of *ZWF1* prevented the increase in redox tolerance in yeast with low PYK activity. (B) ROS levels increase in respiring cells when the PPP is deficient. ROS levels in *ZWF1* and Δ*zwf1* yeast with varying PYK activity were determined with DHE, which preliminarily detects superoxide (left panel), and DCFDA, which detects H_2_O_2_ (right panel). Low PYK activity did not increase DHE and DCFDA oxidation in wild-type, but in Δ*zwf1* yeast. Error bars, ±SD. (C) Low PYK activity increases protein carbonylation in Δzwf1 yeast. Protein extracts (7.5 μg) were analyzed by oxyblotting (left panel) and extracts controlled with Coomassie staining (right panel). *ZWF1* and Δ*zwf1* are juxtaposed images from the same blot/gel. Low PYK activity strongly increased carbonylation in Δ*zwf1* yeast. (D) Mitochondrial damage in *Δzwf1* cells with low PYK activity. PYK models expressing Aco1-eGFP were analyzed for mitochondria morphology. Strains wild-type for *ZWF1* contain typical tubular mitochondria. In combination with low PYK activity, Δ*zwf1* caused mitochondrial fragmentation gradually increasing with low PYK activity; Aco1p indicated 100% mitochondrial network fragmentation in Δ*zwf1 CYC*_pr_-*PYK2* yeast.

To determine if PPP activation is responsible for the increased stress resistance, we deleted *ZWF1* in respiring PYK mutants. *Δpyk1Δpyk2Δzwf1* yeast expressing *TEF_pr_-PYK1* or *CYC_pr_-PYK1* was tested for oxidant resistance. Low levels of *PYK1* increased diamide resistance only in *ZWF1*, but not in *Δzwf1* yeast (Figure 6A). This indicated that oxidative PPP is required in order for PYK to augment oxidative stress resistance.

Then we tested whether the *Δzwf1* deletion also affected ROS levels. ROS levels were measured in logarithmically grown yeast by DHE and DCFDA fluorescence. Similar to Figure 3A, in wild-type *ZWF1* cells, ROS levels did not increase when there was lower PYK activity. However, both stainings detected an increase with respiration when *zwf1* was deleted (Figure 6B). Thus, ROS accumulate in respiring cells only when the oxidative PPP is deficient.

To illustrate consequences on cellular macromolecules, we studied protein carbonylation and mitochondrial morphology. Although no increase in carbonyl levels was observed by oxyblotting in *ZWF1* wild-type cells, these rose upon deletion of Δ*zwf1* (Figure 6C). Thus macromolecules are sufficiently protected from oxidative carbonylation in respiring cells as long as the oxidative PPP is activable. Effects were clearly pictured on the mitochondrial shape. Aco1p tagged with eGFP was chosen as marker for mitochondrial oxidative damage, because it contains an iron-sulfur cluster that is prone for oxidation (Klinger et al., 2010). Aco1-eGFP stained a typical mitochondrial, tubular network in cells with high PYK activity, and when cells were *ZWF1* wild-type. However, depletion of *zwf1* in models with low PYK activity resulted in gradual relocalization. In *Δzwf1 CYC_pr_-PYK2* yeast, the mitochondrial network fragmented to 100% into numerous small roundish mitochondria (Figure 6D). This indicates strong oxidative damage and was previously associated with loss of aconitase activity (Klinger et al., 2010).

## Discussion

Cellular life depends on energy which is shuffled between biochemical reactions in the form of ATP. Its energy charge is maintained by the metabolic network and restored primarily by glycolytic fermentation and respiration (Bolanos et al., 2010; Grüning et al., 2010). Oxidative metabolism is more efficient in producing ATP but produces ROS, such as the superoxide anion in the electron transport chain (Novo and Parola, 2008). In yeast, superoxide preliminary originates from complex III, but also from oxidoreductases which feed the respiratory chain without proton pumping (Nde1, Nde2, and Ndi1) (Luttik et al., 1998). The total rate of ROS production during respiration equals 1%–2% of the metabolized oxygen (Cadenas and Davies, 2000).

Here we show that PYK regulates respiration in *S. cerevisiae.* Yeast express *PYK1* when grown in fermentable carbon sources, where *PYK2* is suppressed (Boles et al., 1997). A switch from *PYK1* to *PYK2*, or simple lowering expression of either isoform, was sufficient to shift from fermentative to oxidative metabolism. Cells with low PYK activity exhibited increased oxygen consumption and displayed growth phenotypes and mRNA expression fingerprints that indicated an increase in mitochondrial energy metabolism.

We were surprised that ROS levels did not increase upon the induction of respiration. ROS oxidize macromolecules (fatty acids, nucleic acids, and proteins). If not properly balanced, the redox state may fall out of equilibrium and cause oxidative or reductive stress. Although oxidative stress is better understood, also excess of reducing equivalents is pathogenic and leads to defects in biochemical reactions, protein folding, and signaling events (Rajasekaran et al., 2007; Tu and Weissman, 2002). As a consequence, respiratory metabolism relies on the capacity of clearing oxidizing molecules, but also on the ability to tune the production of redox equivalents.

We found that the respiring PYK mutants had increased resistance to oxidants. Since clearance of ROS occurs irrespective of the source of free radicals (Apel and Hirt, 2004), this pointed to an increased potential to neutralize superoxide released from the respiratory chain. The grade of resistance to the external stressors varied (Figure 3). Yeast reacts differentially to different oxidants, which depends on the type of free radical released, but also on the oxidant's redox (Nearnst) potential, its primary targets, and different grades of evolutionary adaptation (Thorpe et al., 2004). In this particular case, further differences originate from GAPDH, the TPI neighboring enzyme in glycolysis, which is inactivated by various oxidants to a different extend (Grant et al., 1999) and influences oxidant resistance of yeast with reduced TPI activity (Ralser et al., 2007). As only a marginal fraction of oxidant resistant yeast mutants tolerated a comparably broad spectrum of oxidants in an earlier study (Thorpe et al., 2004), it could be concluded that PYK stimulated a general component of the redox balancing machinery.

A central component of redox metabolism is the PPP. For every glucose equivalent, its oxidative branch reduces two molecules of NADP^+^. In the glutathione system, the primary free radical scavenger, as well as in peroxiredoxin and glutaredoxin systems, NADPH is required to recycle the oxidized form, e.g., to reduce GS-SG to GSH (Grant, 2001; Holmgren et al., 2005). Most mutants of PPP enzymes are sensitive to oxidants (Juhnke et al., 1996), and the NADPH/NADP^+^ ratio collapses when PPP-deficient cells are exposed to H_2_O_2_ (Castegna et al., 2010).

Dynamic PPP activation has been observed upon extracellular addition of oxidants and when cells shift to a nonfermentable carbon source (Cakir et al., 2004; Grant, 2008; Ralser et al., 2007; Shenton and Grant, 2003). This protected cells in two distinct (but overlapping) ways, as it augmented the NADPH/NADP^+^ ratio (Grant, 2008; Ralser et al., 2007) and activated part of the antioxidant gene expression program (Krüger et al., 2011).

In case of exposure to a toxic oxidant dose, PPP activity is rapidly stimulated through oxidative inactivation of glycolytic enzymes (Ralser et al., 2009; Shenton and Grant, 2003). However, there was evidence that this mechanism is not induced by ROS leakage from the respiratory chain: glycolysis is not inhibited during respiration (Meredith and Romano, 1977), redox-prone GAPDH is stable to a continuous oxidant exposure (Cyrne et al., 2010), and even after strong bursts its activity is re-established after a few hours (Colussi et al., 2000). Finally, as shown in this manuscript, ROS levels are not necessarily increased in respiring cells, thus they do not possess a redox state which would trigger oxidative enzyme inactivation.

We discovered that in respiring cells the activity of the oxidative PPP is stimulated through a metabolic feedback loop. PEP accumulated in yeast with low PYK activity and acted as inhibitor of the glycolytic enzyme TPI. This appeared to be a conserved process, as yeast, rabbit, and human TPI were all efficiently inhibited by PEP. Our data do not exclude the possibility that PEP acts also as inhibitor or modulator on other enzymes, but demonstrates that TPI inhibition is sufficient to trigger ROS clearance during oxidative metabolism. We isolated one TPI allele, TPI_Ile170Val_, which was inefficiently inhibited by PEP. This allele has reduced catalytic activity itself, and we have shown earlier that it increases the metabolite content in the PPP (Ralser et al., 2007). When this isoform was expressed, low PYK activity did not further increase oxidant tolerance when PYK activity was lowered.

TPI mutations cause a rare metabolic syndrome, TPI deficiency. Most patients suffering from this rare genetic disease are homozygous, or compound heterozygous for a single TPI allele (TPI_Glu104Asp_) which alters stability and dimer formation (Rodriguez-Almazan et al., 2008). However, the pathogenesis of other alleles is still unknown. The discovery that at least one mutant protein was deficient for PEP feedback inhibition opens a new aspect for research on the pathomechanism, as impaired redox metabolism has been reported as feature of TPI deficiency (Ahmed et al., 2003).

Finally, we investigated whether the PPP is required to clear free radicals upon respiration activation. We prevented the reduction of NADP^+^ in the oxidative PPP by deleting its first enzyme (Zwf1p). Lowering PYK activity did not augment stress resistance in Δ*zwf1* cells. In addition, we used DHE and DCFDA fluorescence to determine ROS levels in respiring PYK mutants. Remarkably, they were unaffected as long as the oxidative PPP was functional, but accumulated in respiring cells upon deletion of Zwf1. Thus, the oxidative PPP is essential for both the increase in oxidant resistance and the stabilization of ROS levels upon the induction of respiration.

**Figure 7:**
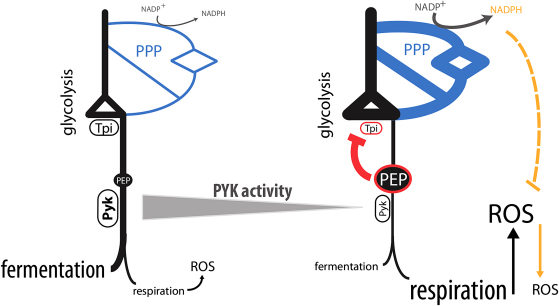
Synchronization of Redox and Energy Metabolism by Pyruvate Kinase Low PYK activity increases respiration. At the same time, the PYK substrate PEP accumulates. This stimulates the PPP by feedback inhibition of TPI, which in turn prevents ROS accumulation during respiration.

These results propose a mechanism for how PYK increases antioxidative capacities (Figure 7). PEP, the PYK substrate, accumulates when the activity of this enzyme is low. This inhibits the glycolytic enzyme TPI. The resulting increase in PPP activity protects cells against oxidants and prevents accumulation of ROS. Recently, it has been reported that PEP in PKM2-expressing cells converts phosphoglycerate mutase (PGM) into a lactate-producing enzyme, which alters glycolysis in proliferating mammalian cells. This may explain the requirement of this feedback loop, as a block of PYK alone would not cause accumulation of upstream metabolites, if the reaction can be surpassed by PGM (Vander Heiden et al., 2010).

PYK-mediated regulation of respiration may differ between yeast and mammalian cells. Consistent with the yeast model are recent investigations demonstrating higher concentration of PKM2 in tumors as in control tissue (Bluemlein et al., 2011), and demonstrating that PKM2 activates fermentative gene expression independent of its activity (Luo et al., 2011). Conversely, however, others have reported increased oxidative phosphorylation with reduced lactate production when PKM maintained high activity (Hitosugi et al., 2009). Further investigations are required to elaborate these yet-unsolved discrepancies in mammalian cells. In this context, both in yeast and mammalian cells, the mechanism for how PYK stimulates oxidative phosphorylation remains to be discovered. We rule out an active role of the PPP, as oxygen uptake also increased upon deletion of PPP enzymes (Supplemental Information, Figure 2). Furthermore, as oxygen uptake of the PYK models increased also on galactose media (Figure 2), a detection of energy shortage by a respective energy sensor falls short in explaining the regulatory mechanism.

However, there is evidence that the metabolic feedback loop presented here is evolutionarily conserved. First, there is evidence that metabolites upstream of PYK accumulate in mammalian cells during the Warburg effect (Vander Heiden et al., 2010) and when PYK is depleted in *B. subtilis* (Emmerling et al., 2002). Second, we have shown here that human and rabbit TPI are more effectively inhibited by PEP compared to yeast TPI (Figure 4B). This could explain our previous observation that transgenic yeast expressing human TPI are more oxidant resistant compared to those expressing yeast TPI (Ralser et al., 2007). Finally, the effect that reduced TPI activity causes an increase in oxidant resistance is conserved, and depletion of Zwf1 (G6PDH) paralogues decreases oxidant tolerance and NADPH in mammalian models (Ho et al., 2000; Ralser et al., 2007; Zhang et al., 2010). Aside the regulation of respiratory energy metabolism, it is assumed that the Warburg effect enables rapidly proliferating tissue to synthesize essential macromolecules (nucleic acids, amino acids, and lipids) from metabolic intermediates, permitting growth and duplication of cellular components during division (Hsu and Sabatini, 2008; Najafov and Alessi, 2010; Vander Heiden et al., 2010). A major fraction of the required intermediates originate from PPP and upper glycolysis; thus metabolic feedback inhibition of TPI by PYK can assure production of these intermediates. Following this line of thought, the feedback loop may represent a therapeutic target because it opens an opportunity to deprive cancer cells from their supply of metabolic intermediates.

## Experimental Procedures

Yeast cultivation, enzyme activity assays, plasmid and yeast strain generation, and qRT-PCR were conducted by standard methods and are available in the Supplemental Information.

### Measurement of Oxygen Consumption, ROS Levels, Carbonylation, and Aconitase 1

Oxygen consumption in exponentially growing yeast cells was determined in an Oxygraph 2k (Oroboros) following the manufacturer's instructions. DHE fluorescence was used to measure ROS (superoxide) as described in Klinger et al. (2010). DCFDA (2′,7′-dichlorofluoresceine diacetate) was used to determine H_2_O_2_ levels in cells grown to midexponential phase in YPD media. DCFDA (10 μM) was added to the cultures for 30 min at 30°C and pellets washed and measured in four replicates in a POLARstar Omega plate reader (BMG Labtech, λex = 490 nm, λem = 524 nm). Protein damage by carbonylation was determined using the OxyBlot Protein Oxidation Detection Kit (Millipore) according to the manufacturer's instructions blotting 7.5 μg protein on PVDF membrane. Aconitase was pictured in yeast cultures transformed with the plasmid pUG35-ACO1. Transformants were diluted from an overnight culture to an OD600 = 0.1 in SC medium lacking uracil and were grown till midexponential phase. Distribution of Aco1-eGFP was pictured with a 100× objective on a Zeiss Axioscope 50 fluorescence microscope.

### Oxidant Tolerance Tests

Oxidant tolerance spot tests were performed as described in (Ralser et al., 2007) and pictures taken after 2–3 days of incubation at 30°C. Quantification of spot growth was achieved via digital image processing using CellProfiler software (Carpenter et al., 2006). In the survival assays, overnight cultures were diluted to an OD600 = 0.1 in YPD and supplemented with diamide to a final concentration of 3 mM or left untreated as control. Of a 1:200 or 1:20000 dilution, 100 μl was plated in triplicates at time points 0 hr and 24 hr onto YPD agar plates. Oxidant resistance in liquid cultures was assayed in replicates of four in 96-well plates. Cells were grown from an OD600 = 0.6 (tert-butyl hydroperoxid), 0.12 (CHP, menadione), or 0.1 (diamide) for 4 hr (menadione, CHP, TBH) or 17 hr (diamide). Growth was measured photometrically in a Spectra Max 250 plate reader (Molecular Devices).

### Metabolite Quantification by MS/MS

Sugar phosphates were quantified by LC-MS/MS as described earlier (Wamelink et al., 2009). In brief, metabolites were extracted in HBSS with 2% perchloric acid, and proteins were precipitated after neutralization with a phosphate buffer. The samples were subsequently supplemented with an internal isotope labeled standard ^13^C_6_-glucose-6P, separated on a water-acetonitrile gradient on a C_18_ RP-HPLC column (LC packings), and analyzed on an API3000 triple quadrupole mass spectrometer (AB/Sciex).

For the determination of PEP, yeast was YPD grown to mid-log phase, centrifuged, washed with water, and frozen at −80°C. To the frozen yeast pellets, glass beads (425–600 μm, Sigma) and 80% methanol in water (300 μl) were added, followed by one cycle on a Fast Prep-24 (MP Biomedicals) for 20 s at 6.5 m/s. Extracts were then cleared by 2× centrifugation at 16,000 g. Quantitative PEP measurements were conducted on a QTRAP5500 hybrid iontrap/triple quadrupole mass spectrometer (AB/Sciex), coupled online to an Agilent 1290 LC system. Separation was achieved on a HILIC column (Acquity BEH HILIC, 1.7 μm, 2.1 × 100 mm [Waters]) by a linear gradient from 100% acetonitrile/ammonium hydrogen carbonate (90/10; A) to 100% acetonitrile/ammonium hydrogen carbonate (50/50; B) between 0.5 and 1 min at a flow rate of 1 ml min^−1^. The gradient was kept at 100% B for 0.5 min before returning to starting conditions. The stop time was set to 3.5 min to allow equilibration of the system prior to the following sample injection. The column temperature was set to 35°C. PEP quantification in yeast extracts was conducted by standard addition.

The MS was run in the negative mode and at a source temperature of 350°C. All other parameters, such as nebuliser and drying gas, influencing the sensitivity of the analysis were optimized prior to the measurements. Quantification of PEP was achieved by monitoring its collision induced (collision energy, −10 V) fragmentation from m/z 167 to 78.8. A detailed protocol will be published elsewhere.

## Supplemental Information

Document S1. Two Figures, One Table, Supplemental Experimental Procedures, and Supplemental References
