# Supplementary material for: Pyruvate Kinase Triggers a Metabolic Feedback Loop that Controls Redox Metabolism in Respiring Cells
Source: Cell Metab. 2011 Sep 7;14(3):415–27. doi: 10.1016/j.cmet.2011.06.017 (PMC3202625; doi:10.1016/j.cmet.2011.06.017)
Supplement: Document S1. Two Figures, One Table, Supplemental Experimental Procedures, and Supplemental References [file mmc1.pdf]

Cell Metabolism, Volume *14*

Supplemental Information

Pyruvate Kinase Triggers a Metabolic Feedback Loop that Controls Redox Metabolism in Respiring Cells

Nana-Maria Grüning, Mark Rinnerthaler, Katharina Bluemlein, Michael Mülleder, Mirjam M.C. Wamelink, Hans Lehrach, Cornelis Jakobs, Michael Breitenbach, and Markus Ralser

<u>**Inventory of Supplemental Information**</u>

- **Figure S1**
  Legend Figure S1

  Figure S1 is connected to Figure 3B. Figure S1 demonstrates an increase of resistance to the oxidant diamide with decreased PYK activity also on SC media containing galactose as carbon source.

- **Table S1**
  Legend Table S1

  Table S1 is connected to Figure 5B and presents absolutes values of PPP metabolites which are shown as ratios in Figure 5B.

- **Figure S2**
  Legend Figure S2

  Figure S2 is connected to the discussion part of the manuscript (Figure 7). The increase in oxygen consumption in PPP and *PCK* mutants with low PYK activity rules out a regulatory role of these enzymes in respiration activation.

- **Supplemental Experimental Procedures**
  Yeast cultivation
  Plasmids
  Gene deletion
  Bona fide ρ0
  Quantitative RT-PCR
  Enzyme activity assays

- **Supplemental References**

Figure S1

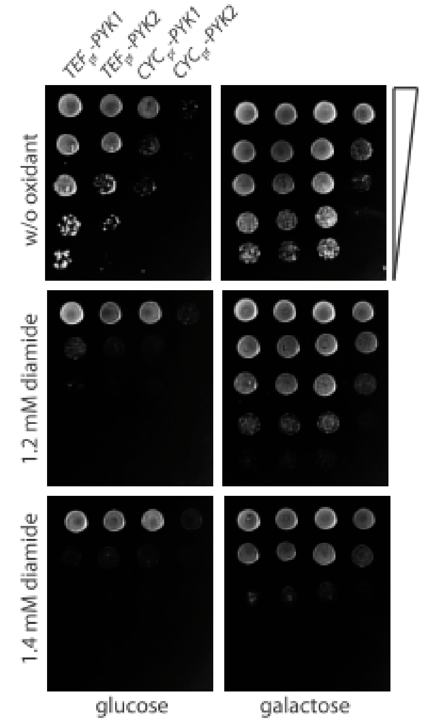

Figure S1, related to Figure 3B: Reduced PYK activity and galactose media leads to higher resistance to oxidants. Strains were grown over night, diluted to an OD_{600} of 3.0, and spotted as 1:5 dilution series onto YPD and YPGal containing diamide at the indicated concentration.

Table S1

		g6p / f6p	6pg	r5p	x5p / rib5p	s7p	e4p	dhap	gly3p	
glucose	BY4741 wt	0,685	0,075	0,117	0,033	0,080	0,050	0,760	0,011	mM / (ml*OD600)
		100,00	100,00	100,00	100,00	100,00	100,00	100,00	100,00	relative concentration (%)
	TEFpr-PYK1	0,854	0,087	0,154	0,029	0,073	0,058	0,979	0,014	
		124,61	115,70	131,17	89,73	91,67	115,69	128,81	130,07	
	TEFpr-PYK2	1,028	0,107	0,233	0,039	0,129	0,085	1,252	0,027	
		150,09	142,41	198,32	118,80	160,74	170,56	164,75	252,24	
	CYCpr-PYK1	1,329	0,113	0,211	0,039	0,096	0,095	1,567	0,023	
		194,10	150,69	180,00	120,46	120,01	190,93	206,15	213,50	
galactose	BY4741 wt	3,949	0,177	0,161	0,059	0,118	0,120	1,081	0,026	
		100,00	100,00	100,00	100,00	100,00	100,00	100,00	100,00	
	TEFpr-PYK1	3,973	0,149	0,226	0,055	0,141	0,098	1,266	0,026	
		100,61	84,46	140,20	93,20	119,77	81,82	117,16	100,52	
	TEFpr-PYK2	4,280	0,177	0,234	0,074	0,151	0,109	2,296	0,048	
		108,37	99,99	145,51	124,65	128,22	90,37	212,50	181,65	
	CYCpr-PYK1	4,899	0,259	0,199	0,117	0,188	0,115	3,135	0,054	
		124,04	146,85	123,37	197,37	159,30	95,91	290,09	205,26	
	CYCpr-PYK2	4,988	0,434	0,381	0,195	0,253	0,201	5,825	0,095	
		126,31	245,83	236,48	329,13	215,17	167,25	539,02	360,52	

Table S1, related to Figure 5B: PPP metabolites are increased in strains with low PYK activity. Values are given normalized to dhap concentration in a reference measurement (correction factor 36.5, black) and as ratio to metabolite concentration in the BY4741 wild-type strain (grey). g6p (glucose 6-phosphate), f6p (fructose 6-phosphate), 6pg (6-phospho gluconate), r5p (ribose 5-phosphate), x5p (xylulose 5-phosphate), rib5p (ribulose 5-phosphate), s7p (sedoheptulose 7-phosphate), e4p (erythrose 4-phosphate), dhap (dihydroxyacetone phosphate), gly3p (glyceraldehydes 3-phosphate).

Figure S2

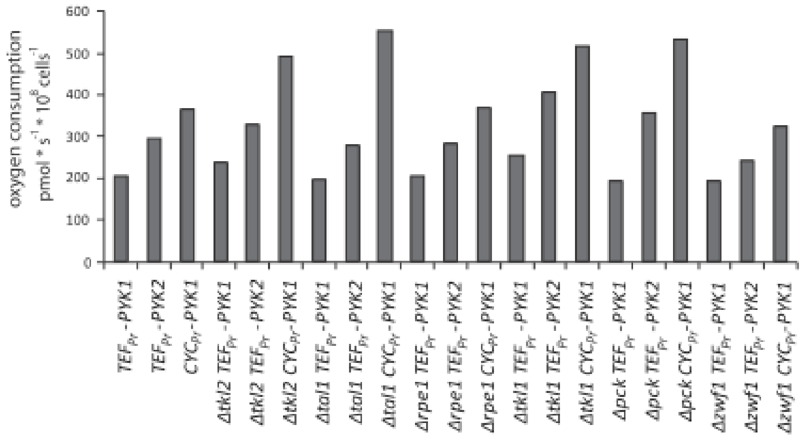

Figure S2, related to discussion/Figure 7: Oxygen consumption increases in with low PYK activity in PPP and *PCK* mutants. PPP enzymes or *PCK1 were deleted in ∆pyk1∆pyk2* yeast strains containing the different PYK constructs. Oxygen consumption was determined for logarithmically YPD -growing cultures in a closed chamber oxygraph (Oroboros). Tkl2 (encoding transketolase), Tal1 (transaldolase), Rpe1 (ribulose 5-phosphate epimerase), Tkl1 (transketolase), Pck (pyruvate carboxykinase), Zwf1 (glucose 6-phosphate dehydrogenase). Low PYK activity increased oxygen consumption in all deletion strains tested.

Supplemental Experimental Procedures

Yeast cultivation

Yeast were grown at 28-30°C either in yeast-extract peptone 2% dextrose (YPD), yeast-extract peptone 2% galactose (YPGal), 3% ethanol/0.1% glucose (YPEtOH) or in synthetic complete (SC) media lacking the indicated amino acids/bases.

Plasmids

Plasmids encoding TPI were previously described (Ralser et al., 2006). PYK-encoding plasmids were generated by amplifying *PYK1* and *PYK2* from yeast genomic DNA by PCR, and ligating the products into centromeric yeast plasmids containing the *TEF1* promoter (p413TEF), the *CYC1* promoter (p413CYC), or a *GPD1* promoter (p416GPD) (Mumberg et al., 1995). All plasmids were verified by sequencing and primer sequences are given in the table below.

PYK1-fw-BamH1	5'-GAGGATCCATGTCTAGATTAGAAAGA-3'
PYK1-as-Sal	5'-GAGTCGACTTAAACGGTAGAGACTTG-3'
PYK2-fw-BamH1	5'-GAGGATCCATGCCAGAGTCCAGATTG-3'
PYK2-as-Sal1	5'-GAGTCGACCTAGAATTCTTGACCAAC-3'
underlined DNA sequences indicate introduced restriction sites	

Gene deletion

Genes were deleted in BY4741 strains by homologous recombination, by single gene replacement with the nourseothricin (natMX4), kanamycin (kanMX4), or hygromycin (hphMX4) markers. Primer pairs (which overlap with 20 bases of the marker gene and 35-45 bases with the target locus) were used to amplify the marker cassette and then transformed into yeast. Positive transformants were selected on YPD containing antibiotics, and isolated recombinants were verified by PCR. Primer sequences are given in the table below.

MX4 deletion cassettes for PYK1 fwd	ATTTACAAGACACCAATCAAAACAAATAA AACATCATCACAAGCTTGCCTTGTCCCCGCCG
MX4 deletion cassettes for PYK1 rev	TTAAACGGTAGAGACTTGCAAAGTGTTGG AGTGACCAGCATCGACACTGGATGGCGGCGT
MX4 deletion cassettes for PYK2 fwd	CCTCTACGTCCATTGTAAGATTACAACAAA AGCACTATCGAGCTTGCCTTGTCCCCGCCG
MX4 deletion cassettes for PYK2 rev	TACTAGAATTCTTGACCAACAGTAGAAATG CGTAAGGTATTCGACACTGGATGGCGGCGT
underlined DNA sequences indicate introduced restriction sites	

Isogenic PYK mutants were generated by plasmid shuffling**.** *Δpyk2* yeast was transformed with an *URA3*-plasmid encoding for PYK1 (p416GPD-PYK1). Then, endogenous *PYK1* was deleted using *natMX4*, and positive knock-outs were selected by PCR. The *Δpyk1Δpyk2* pCEN-*URA3-PYK1* strain was subsequently transformed with HIS3-marked PYK plasmids (*p413TEF-PYK1, p413CYC-PYK1, p413TEF-PYK2, p413CYC-PYK2*). Finally, the *URA3*-plasmid was counter-selected for positive transformants on SC^{-HIS} containing 0.15% 5'FOA. *Δpyk1Δpyk2Δzwf1* and *Δpyk1Δtpi1* yeast expressing *PYK1*, *PKY2*, and/or TPI from centromeric plasmids were generated in a similar fashion.

Bona fide ρ0 strains were generated through repeated treatment with 50 µg/ml ethidium bromide as previously described (Goldring et al., 1970).

Quantitative RT-PCR

qRT-PCR was performed as previously described in (Wamelink et al., 2010). Yeast were cultivated overnight in YPD, washed once in water, and grown to log phase (OD_{600} ~ 0.8) in YPD or YPGal. For boost experiments, YPD was exchanged with YPGal one hour before harvesting cells. mRNA was extracted and qRT-PCR was performed using an ABI prism 7800HT system. Primer sequences are listed in the Supplemental Information. Expression of

COX1, COX2, COX3 was normalized to the expression of the reference genes *ATG27* and *TAF10* as by the method of (Pfaffl, 2001).

Enzyme activity assays

Pyruvate kinase activity was determined as described by (Bergmeyer et al., 1974). Briefly, a reaction mixture containing 24 mM KH_2PO_4/K_2HPO_4 (pH 7.0), 150 µM NADH, 1 mM fructose 1,6 bisphosphate, 2.4 mM ADP, 25 U lactate dehydrogenase (Sigma-Aldrich), 10 mM $MgSO_4$, and 4 µg centrifugation-cleared whole-cell extract was supplemented with 800 µM PEP. OD_{340} was used to detect NADH oxidation in 6- to 10-s intervals using an spectrophotometer (Amersham US 2000). TPI activity was determined as previously described (Ralser et al., 2006). K_m and K_i were determined by saturation curves with gly3p and PEP, respectively, in yeast extracts (BY4741), transgenic yeast expressing human TPI (MR101) (Ralser et al., 2006), or purified rabbit muscle TPI (Sigma-Aldrich).

Supplemental References

Bergmeyer, H.U., Gawehn, K., and Grassl, M. (1974). Methods of Enzymatic Analysis, 2nd Edition, Academic press, NY *1*, 509-510.

Mumberg, D., Muller, R., and Funk, M. (1995). Yeast vectors for the controlled expression of heterologous proteins in different genetic backgrounds. Gene *156*, 119-122.

Pfaffl, M.W. (2001). A new mathematical model for relative quantification in real-time RT-PCR. Nucleic Acids Res *29*, e45.

Wamelink, M.M., Gruning, N.M., Jansen, E.E., Bluemlein, K., Lehrach, H., Jakobs, C., and Ralser, M. (2010). The difference between rare and exceptionally rare: molecular characterization of ribose 5-phosphate isomerase deficiency. J MolMed *88*, 931-939.
